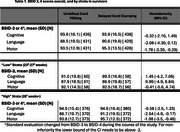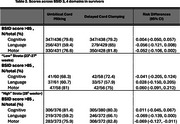# Oral Concurrent Session 3 – Fetus and Fetal Intervention: Wednesday, February 11, 2026 1:00 PM – 3:00 PM

**DOI:** 10.1002/pmf2.70165

**Published:** 2026-02-09

**1:00 PM  –  1:15 PM**

## 29 | Childhood Obesity and Other Outcomes in Offspring Exposed to Adjunctive Azithromycin From the C/SOAP Trial

Akila Subramaniam^1^; Justin M. Leach^1^; Jeff M. Szychowski^2^; Alan T. Tita^1^

On behalf of the C/SOAP Childhood Follow‐up Study Consortium

^1^Center for Research in Women's Health, University of Alabama at Birmingham, Birmingham, AL; ^2^Department of Biostatistics, University of Alabama at Birmingham, Birmingham, AL

**Objective**: In the Cesarean Section Optimal Prophylaxis (C/SOAP) trial, azithromycin (AZI) reduced puerperal infections by 50% compared to placebo (patients in both groups received standard cesarean antibiotic prophylaxis). However, concerns about AZI's effect on the neonatal microbiome and thus, long‐term childhood health persist. As such, we compared childhood health outcomes in offspring from the C/SOAP trial after perinatal exposure to AZI versus placebo.

**Study Design**: We performed a non‐inferiority (NI) prospective follow‐up study (from Jan 2021–July 2025) assessing health in children aged 7–14 of women enrolled in the original C/SOAP trial (2011–14). Children were compared by exposure to AZI or placebo. Our primary outcome was childhood obesity (BMI ≥95th percentile for age/sex). Secondary outcomes included select adverse respiratory and gastrointestinal (GI) outcomes. Risk differences (RD), with 95% confidence intervals [CI] were calculated and evaluated using a NI margin (NIM) of 10%. Multivariable analyses were planned a priori to adjust for known potential confounders.

**Results**: Of the 2013 participants from C/SOAP, 1990 were eligible for follow‐up, and 1024 were successfully contacted. Of those contacted, 505 children of these participants (49.3%) enrolled: 255 (50.5%) exposed to AZI and 250 (49.5%) placebo. Characteristics were similar between groups (Table 1). The rate of childhood obesity in the AZI group was non‐inferior (adjusted RD −10.4% [−20% to −0.8%]; [−0.8% <NIM 10%]) and potentially lower than placebo (26.7% AZI vs 34.4% placebo). Non‐inferiority was also observed for almost all secondary outcomes (upper CI bounds <10% NIM, Table 2). Of note, microbiome‐mediated GI disorders were significantly lower in the AZI group.

**Conclusion**: In this C/SOAP follow‐up study, adjunctive AZI for cesarean prophylaxis was non‐inferior to placebo in terms of rate of childhood obesity and other microbiome‐mediated disorders. Despite the low follow‐up and pending respiratory (spirometry) and neurodevelopment data; these findings are reassuring regarding child health outcomes after perinatal exposure to AZI.

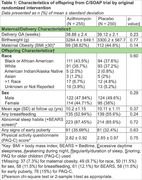

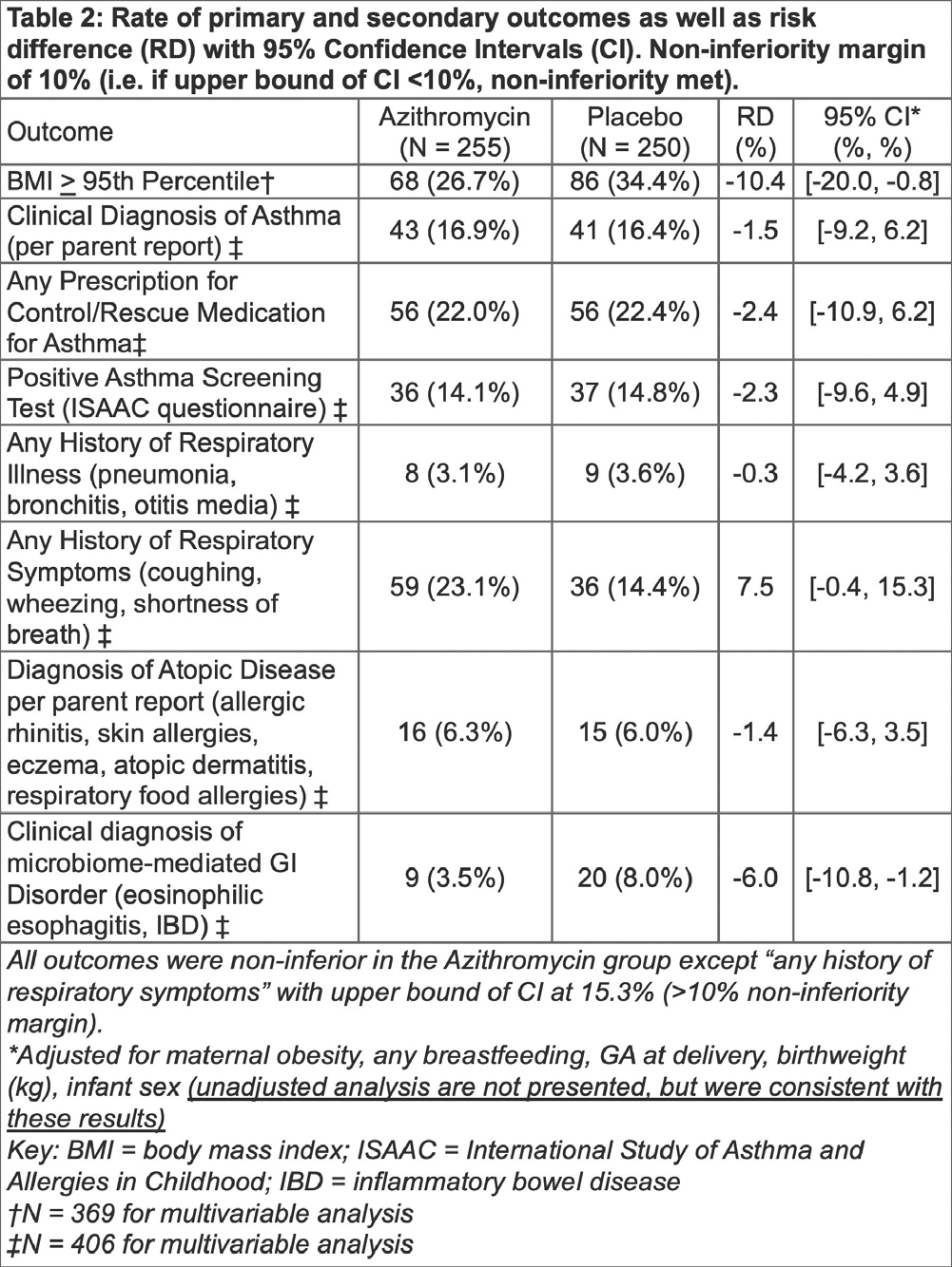

**1:15 PM  –  1:30 PM**

## 30 | Prenatal Surgical‐to‐Delivery Interval and 30‐Month Neurodevelopment: A Secondary Analysis of the MOMS Cohort

Nikan Zargarzadeh^1^; Claudio V. Schenone^2^; Eyal Krispin^2^; Jonathan Castillo^3^; Heidi Castillo^3^; Alireza A. Shamshirsaz^4^; Ashish Premkumar^5^

^1^Boston Children's Hospital, Boston, MA; ^2^Boston Children's Hospital, Harvard Medical School, Boston, MA; ^3^UNMC Division of Developmental Medicine, Omaha, NE; ^4^Boston Children's Hospital, Brigham and Women's Hospital, Beth Israel Deaconess Medical Center, Harvard Medical School, Boston, MA; ^5^University of Chicago, Chicago, IL

**Objective**: To evaluate the impact of the prenatal surgical‐to‐delivery interval—the time between prenatal fetal myelomeningocele (fMMC) repair and delivery—on childhood neurological outcomes up to 30 months of life (MOL).

**Study Design**: This was a secondary analysis of the prenatal fMMC repair arm of the Management of Myelomeningocele Study using data provided by the National Institutes of Health (NIH). The primary exposure was the surgery‐to‐delivery interval, dichotomized into the lowest quartile (58.2 days) and all others (58.2–110 days). The outcomes were perinatal death, need for shunt by 12 months of life (MOL), walking independently, the difference between anatomic and motor levels, Bayley II, Peabody, and WeeFIM scores. Bivariate analyses were performed using Fisher's and Wilcoxon tests. Linear regression was used to assess associations between surgical‐to‐delivery interval and Bayley scores, with adjustment for covariates.

**Results**: 90 participants were available for analysis, *n* = 23 (25.5%) in the lowest quartile and *n* = 67 (74.5%) in the higher quartiles. At 30 months, children in the shorter interval group had lower Bayley mental (82.9 [14.7] vs. 91.4 [14.7], *p* = 0.02) and psychomotor scores (54.6 [11.2] vs. 67.2 [17.9], *p* = 0.002), worse motor function relative to lesion level (*p* = 0.02), and were less likely to walk independently (23.8% vs. 50.8%, *p* = 0.05). After adjusting for preoperative ventricular size and accounting for collinearity with gestational age via residual analysis, longer surgical‐to‐delivery interval remained significantly associated with higher Bayley mental scores (adjusted coefficient: 0.24 [95% CI: 0.07, 0.41], *p* = 0.005).

**Conclusion**: A shorter interval between prenatal fMMC repair and delivery is associated with worse neurodevelopmental outcomes in some domains at 30 months. Notably, this association persists even when accounting for gestational age at delivery, indicating that the duration of pregnancy *following* fetal surgery exerts an independent effect. These findings emphasize the dual importance of prolonging gestation after intervention and carefully selecting the timing of surgery.

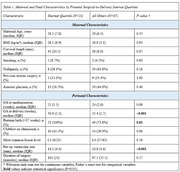

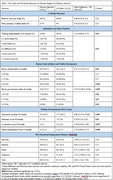

**1:30 PM  –  1:45 PM**

## 31 | **AI‐Powered Fetal Movement Detection via Smartphones With the Potential to Reduce Risk of Late Stillbirth**

Anna Madden‐Rusnak^1^; Kenneth J. Moise, Jr.^1^; Kelly P. Gaither^2^; Kathy J. Lowry^1^; Emily Hutson^1^; Danielle Bruns^1^; Reinaldo Valero^1^

^1^Department of Women's Health, Dell Medical School at the University of Texas at Austin, Austin, TX; ^2^Texas Advanced Computing Center, Austin, TX

**Objective**: Reducing late‐term stillbirth remains challenging due to costly fetal monitoring and unreliable patient perception of fetal movement. This study explores a low‐cost approach using smartphone audio recordings and Artificial Intelligence (AI) to provide objective, reliable fetal movement (FM) metrics.

**Study Design**: This longitudinal cohort study enrolled pregnant patients (≥22 weeks GA) in a Southern US city for up to five 30‐minute sessions each. Fetal movements (gross, breathing, hiccups) were recorded via smartphone audio from the abdomen, ultrasound, and patient perception. Pre‐processed acoustic features were analyzed to assess effects of BMI and GA. PERMANOVA compared GA groups (early: 22–27 w; middle: 28–32 w; late: ≥33 w) and BMI categories (normal <25; overweight 25–29.9; obese ≥30). The AI model combined machine learning and deep learning, using gradient boosting to improve FM classification. Ultrasound‐detected FMs (total and average per session) and AI vs patient accuracy are reported by FM type.

**Results**: Thirty patients (87% White, 53% multiparous) with singleton pregnancies were enrolled. Most (70%) completed all 5 visits (range 3–5), totaling 2070 minutes of data. GA and BMI significantly affected audio features (*p* < 0.001), with stronger GA effects between early and middle groups. BMI remained mostly stable across GA within patients, but acoustic features differed across BMI categories. AI models outperformed patient perception for all fetal movement types: ∼5x more accurate for gross movements, ∼24x for breathing, and ∼3x for hiccups.

**Conclusion**: AI detection of FM from smartphone audio is both feasible and more reliable than patient perceptions. Given the ubiquity and affordability of smartphones, this approach offers a scalable, low‐cost solution for at‐home fetal monitoring. This approach provides an objective tool to detect changes in fetal activity that may precede late stillbirth. By leveraging accessible devices, this application holds promise for reducing disparities in prenatal care access, particularly in underserved or resource‐limited populations.

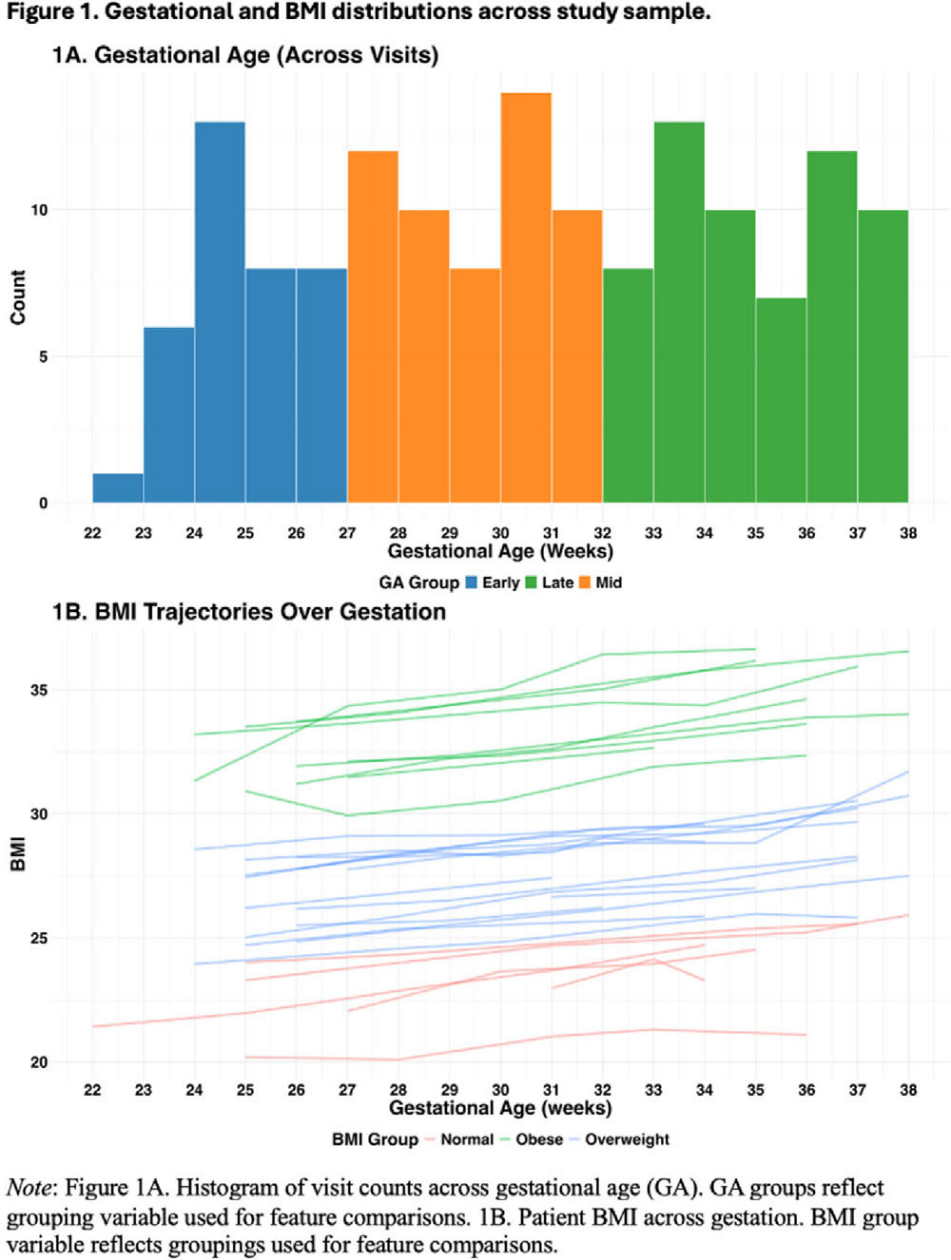

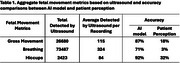

**1:45 PM  –  2:00 PM**

## 32 | **Antenatal Risk Factors for Neonatal Hypoxic‐Ischaemic Encephalopathy: A Prospective National Population‐Based Case‐Control Study**

Pierre Delorme^1^; Rayan Hamadmad^2^; Loïc Sentilhes^3^; Hugo Madar^3^; Isabelle Guellec^4^; Antoine Villotitch^5^; Camille Le Ray^6^; Thierry Debillon^7^; Anne Ego^5^; Gilles Kayem^8^

^1^Sorbonne Université/Department of Obstetrics and Gynecology, Trousseau Hospital, AP‐HP, sorbonne université, Ile‐de‐France; ^2^Sorbonne Université, sorbonne universite, Ile‐de‐France; ^3^Bordeaux University Hospital, Bordeaux Universitary Hospital, Aquitaine; ^4^CHU de Nice ‐ Hôpital l'Archet, CHU DE NICE HOPITAL DE LARCHET, Provence‐Alpes‐Cote d'Azur; ^5^Univ. Grenoble Alpes, CNRS, Public Health Department CHU Grenoble Alpes, Grenoble, Univ. Grenoble Alpes, CNRS, Public Health Department CHU Grenoble Alpes, Grenoble, Auvergne; ^6^Department of Obstetrics and Gynecology, Port‐Royal Maternity Hospital, AP‐HP, Cochin Hospital, FHU PREMA, Paris, France, Paris, Ile‐de‐France; ^7^Neonatal Intensive Care Unit, Grenoble Alpes University Hospital, Grenoble, France, Neonatal Intensive Care Unit, Grenoble Alpes University Hospital, Grenoble, France, Auvergne; ^8^Trousseau Maternity, Paris, Ile‐de‐France

**Objective**: Hypoxic‐ischaemic encephalopathy (HIE) remains a leading cause of neonatal mortality and long‐term neurological disability. This study aimed to identify maternal and perinatal factors associated with moderate‐to‐severe HIE using national, prospective, population‐based data.

**Study Design**: Population‐based case‐control study using prospectively collected data.

Sixty‐eight level III neonatal intensive care units (NICUs) in France (except one region) for cases; all levels of maternity units for controls.

Adjusted odds ratios (aORs) with 99% confidence intervals (CIs), estimated using mixed‐effect logistic regression models using multiple imputation for missing data.

**Results**: 792 neonates with HIE from the LyTONEPAL cohort (2015–2017), including all live births at ≥34 weeks’ gestation admitted to participating NICUs, and 11,095 controls from the 2016 French national perinatal survey. Most neonates with HIE were born by caesarean delivery (62.4%). A sentinel event was identified in 34%, most frequently placental abruption (10.9%) and uterine rupture (6.7%). Fetal bradycardia without an identifiable sentinel event occurred in 19.5%. HIE was independently associated with maternal age ≥35 years (aOR 1.53), obesity (BMI ≥30, aOR 1.71), and pre‐existing diabetes (aOR 2.57). Birth in sub‐Saharan Africa (aOR 2.11) or other non‐European countries (aOR 1.89) was also associated with higher risk. HIE risk followed an inverted J‐shaped distribution across gestational age, with lowest risk at 39–39+6 weeks. Risk was increased before 37 weeks (aOR 5.43), at 37–37+6 weeks (aOR 2.78), and at ≥41 weeks (aOR 1.41). Birth weight <10th percentile increased risk (aOR 2.46), while antenatal suspicion of growth restriction was protective (aOR 0.55).

**Conclusion**: This national study identified maternal, fetal, and gestational factors independently associated with HIE. Several potentially modifiable or detectable risk factors—including obesity, preterm, early or late‐term birth, and missed antenatal detection of growth restriction—highlight opportunities to improve perinatal surveillance and intrapartum care.

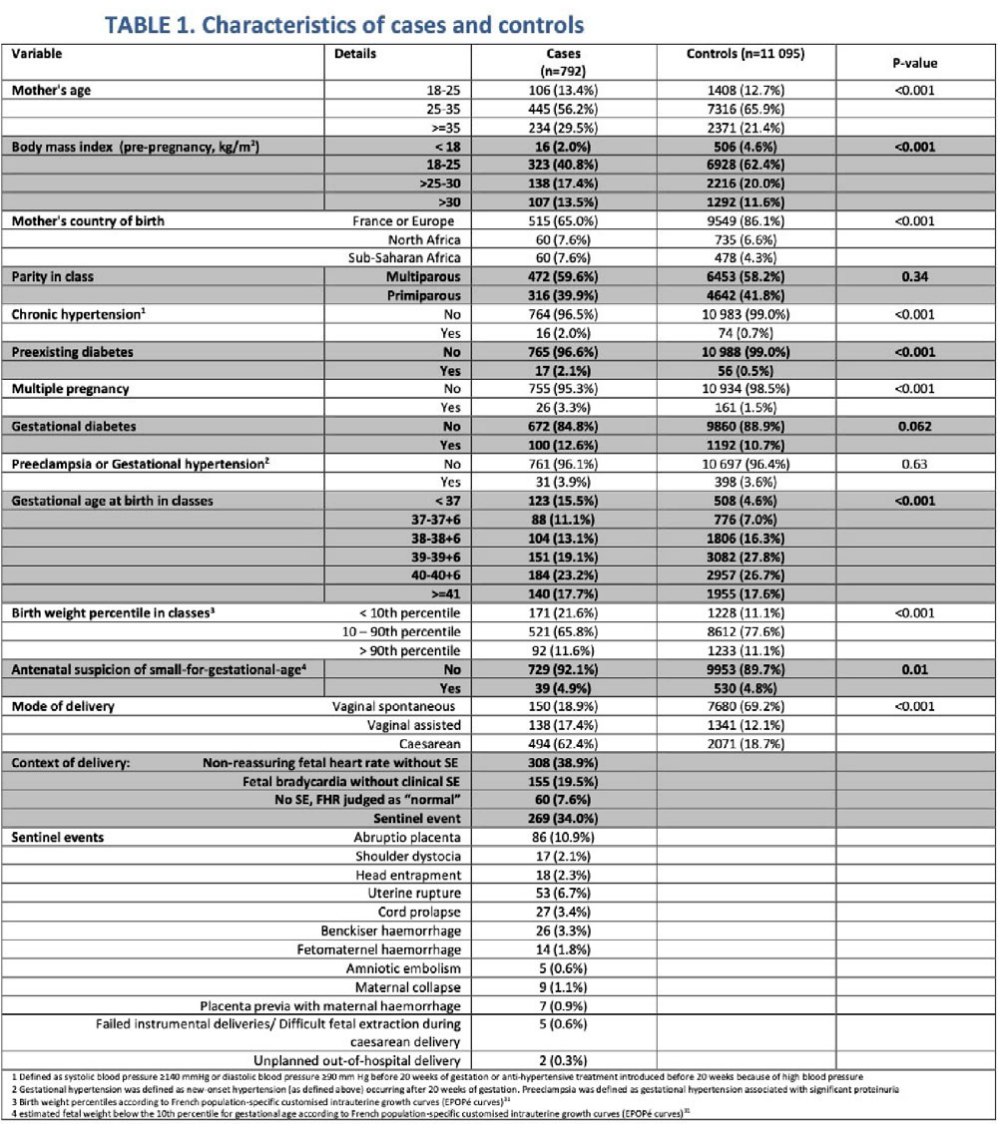

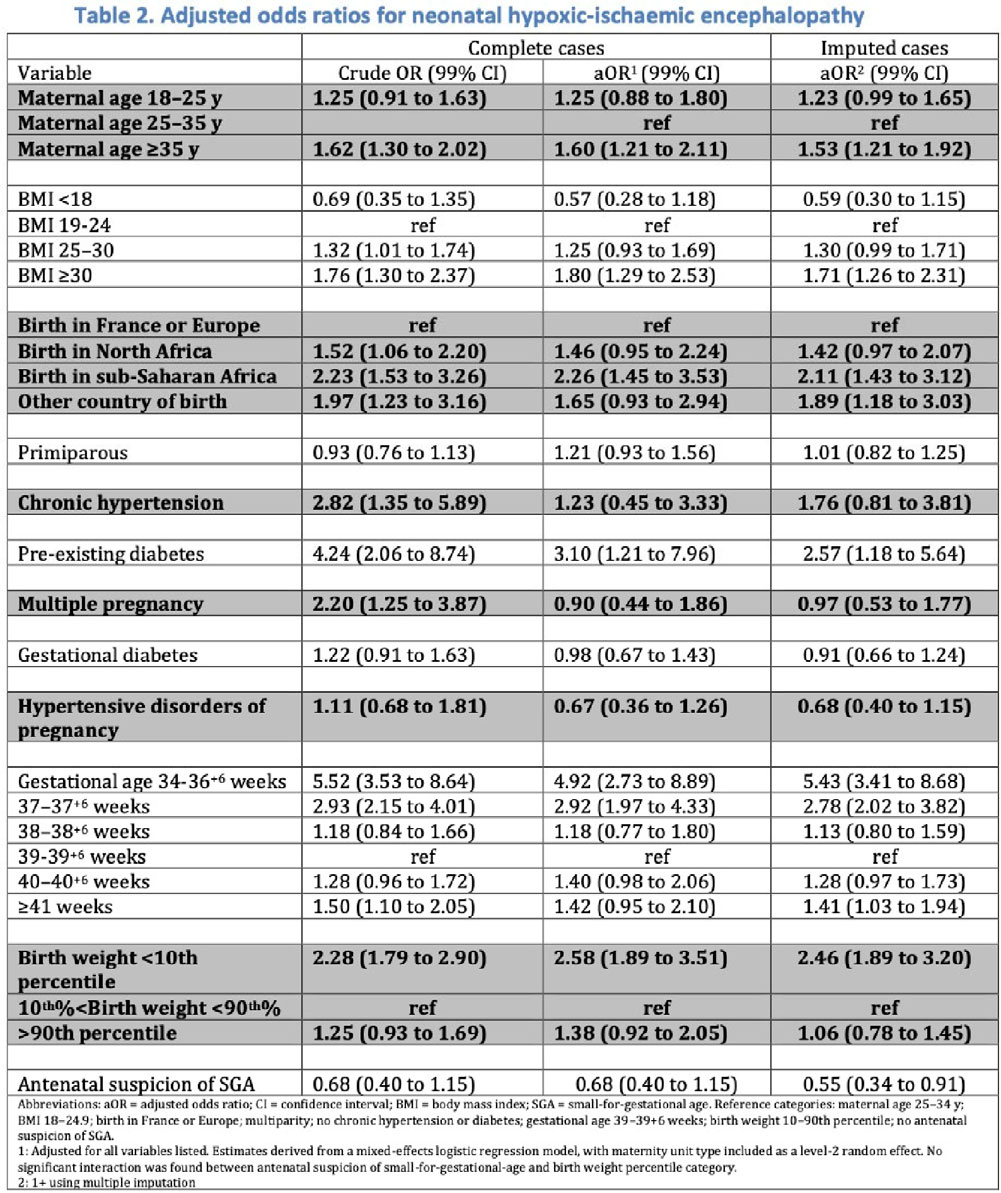

**2:00 PM  –  2:15 PM**

## 33 | **Fetal Hematologic Profile in Monochorionic Twins With Anemia‐Polycythemia Sequence Undergoing Serial Transfusion and Partial Exchange**

Alexandra D. Forrest^1^; Jena L. Miller^2^; Michelle Kush^3^; Mara Rosner^2^; Ahmet A. Baschat^4^

^1^Johns Hopkins University, Baltimore, MD; ^2^Johns Hopkins Center for Fetal Therapy, Johns Hopkins University/Baltimore, MD; ^3^Johns Hopkins Center for Fetal Therapy, Johns Hopkins University/BAltimore, MD; ^4^Johns Hopkins University, Johns Hopkins University, MD

**Objective**: Unequal red blood cell sharing in monochorionic twins with anemia‐polycythemia sequence (TAPS) results in donor anemia and recipient polycythemia. Intrauterine transfusion (IUT) in the donor with or without partial exchange transfusion (PET) in the recipient is one treatment option. The fetal hematologic profile in TAPS has not been well characterized. We aimed to evaluate hematologic findings at diagnosis and in response to serial transfusions.

**Study Design**: We conducted a single‐center retrospective cohort study of fetuses with TAPS treated with IUT and PET. Fetal blood samples were analyzed at each procedure for hemoglobin, hematocrit, reticulocyte count and fraction, immature reticulocyte and platelet fractions, platelet count, and nucleated red blood cells. Initial donor and recipient values were compared and followed longitudinally.

**Results**: 11 patients with stage 2 TAPS underwent 150 procedures (78 donor IUTs, 71 recipient PETs, 1 blood sampling). Median gestational age at first procedure was 27 + 3 weeks (range 23 + 6–30 + 0) and at delivery 32 + 3 weeks (29 + 5–34 + 0), prolonging pregnancy by a median of 35 days (range 5–66). Donors had significantly lower hemoglobin, hematocrit, and platelets, and higher reticulocyte and immature platelet fractions than recipients (all *p* < 0.01). Some recipients had incomplete suppression of reticulocyte production. Serial transfusions were more effective in correcting donor hematologic abnormalities than they were in addressing those observed in the recipient.

**Conclusion**: In this large series of fetal blood sampling in TAPS, donors demonstrated increased hematopoiesis with associated thrombocytopenia while polycythemic recipients largely exhibited downregulated erythropoiesis. The findings point to complex hematopoietic impacts of TAPS that may involve shared platelet and red‐cell precursors in donors and paradoxical hematopoiesis in the recipient possibly due to transfusion of erythropoietin. These findings underscore the asymmetric physiology of TAPS and support serial transfusions as a strategy to prolong pregnancy in cases diagnosed beyond 26 weeks.

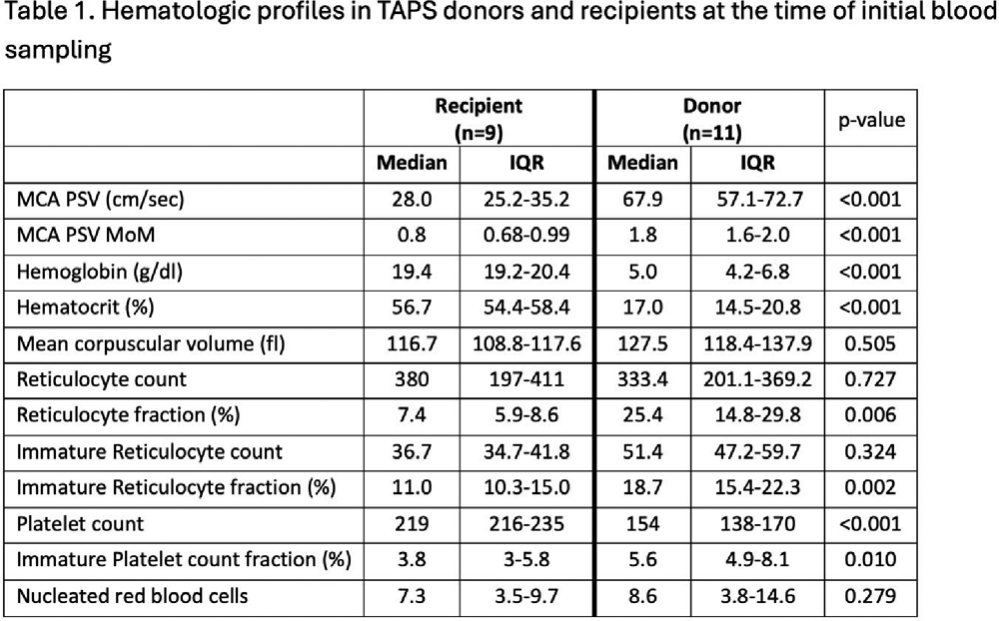

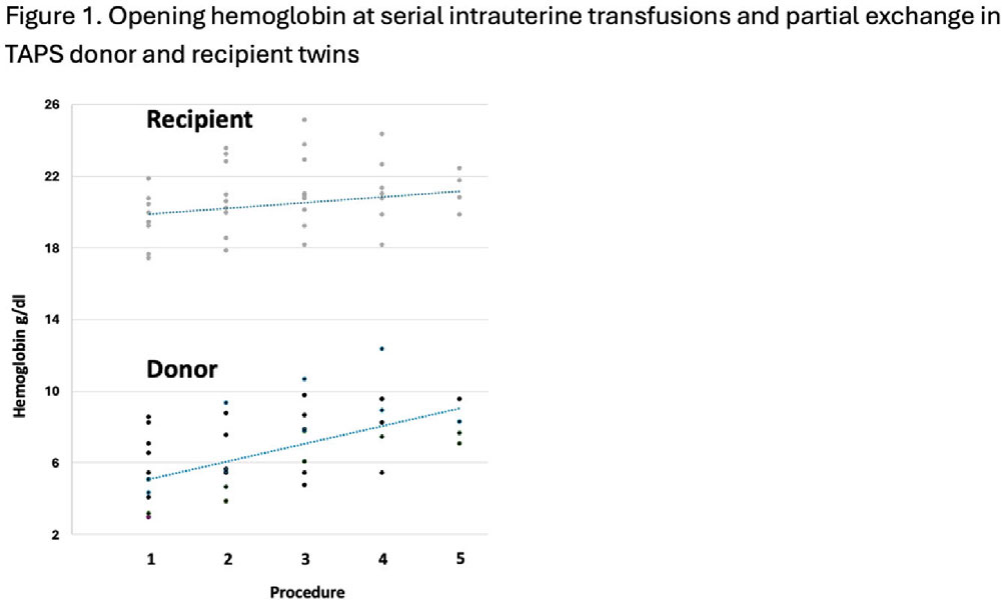

**2:15 PM  –  2:30 PM**

## 34 | **Perioperative Middle Cerebral Artery Flow Changes in Twin‐Twin Transfusion Syndrome and 2‐Year Neurodevelopmental Outcome**

Ramen H. Chmait^1^; Lisa M. Korst^2^; Arlyn S. Llanes^1^; Moshe Fridman^3^; Adam Beasley^4^; Douglas L. Vanderbilt^4^

^1^Keck School of Medicine of USC, University of Southern California, Keck School of Medicine of USC, University Of Southern California, CA; ^2^Childbirth Research Associates, North Hollywood, CA; ^3^AMF Consulting, Los Angeles, CA; ^4^Children's Hospital Los Angeles, Los Angeles, CA

**Objective**: For twin‐twin transfusion syndrome (TTTS) patients who underwent fetoscopic laser surgery, we hypothesized that a rise in the postoperative middle cerebral artery peak systolic velocity multiples of the median (MCA‐PSV MoM) would later be associated with lower Battelle Developmental Inventory 2^nd^ Edition (BDI‐2) scores because acute fetal blood loss may lead to developmental abnormalities.

**Study Design**: In this retrospective cohort study of laser‐treated TTTS patients, the change in fetal MCA‐PSV MoM (Delta) was calculated as the postoperative (∼24 hours after laser surgery) minus preoperative value; a positive Delta signifies a postoperative rise (suggestive of acute blood loss). The outcomes were the Total BDI‐2 score (BDI Total) and the individual domain scores (ie, Adaptive, Cognitive, Communication, Motor, Personal/Social) at 2 years of age, standardized to a mean (SD) of 100 (15). For each outcome, patient characteristics were tested independently in multilevel linear regression models to identify relevant covariates; backward elimination was then applied to the full model.

**Results**: BDI‐2 evaluation was conducted for 99 children (56 families), with median (range) for BDI Total 101 (58 to 132), BDI Motor 108 (55 to 127), and Delta 0.020 (−0.84 to 0.78). In bivariate analysis with the outcome BDI Total, the regression coefficient (β) for the Delta was β = −5.21 (−12.25 to 1.82), *p* = .144; upon adjustment, β = −6.84 (−13.60 to −0.07), *p* = .048 (ie, for every 0.1‐point increase in Delta, the BDI Total score dropped 0.7 points) (Table 1). The Delta was associated with the Motor domain: β = −8.98 (−15.61 to −2.36), *p* = .009; upon adjustment, β = −10.18 (−16.25 to −4.12), *p* = .001 (ie, for every 0.1‐point increase in Delta, the BDI Motor score dropped 1.0 points) (Table 2).

**Conclusion**: A rise in the postoperative MCA‐PSV MoM was associated with a score decrease in the total BDI, and specifically, the Motor domain scores. Further exploration of this relationship appears warranted.

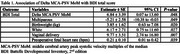

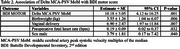

**2:30 PM  –  2:45 PM**

## 35 | **Postoperative Fetal Imaging is Predictive of Need for CSF Diversion After Fetal Spina Bifida Repair**

Katherine H. Bligard^1^; Jesse Vrecenak^1^; Jennifer M. Strahle^2^; Sasidhar Karuparti^1^; Jagruti Anadkat^1^; Nandini Raghuraman^3^; Jeannie C. Kelly^3^; Sherri Jackson^3^; Bree A. Goodman^2^

^1^Washington University School of Medicine, Saint Louis, MO; ^2^Washington University School of Medicine, St. Louis, MO; ^3^Washington University School of Medicine, Washington University School of Medicine, MO

**Objective**: Fetal surgery for open neural tube defects (ONTDs) reduces but does not eliminate the need for cerebrospinal fluid (CSF) diversion in the first year of life. While some preoperative factors including lateral ventricle (LV) width have been linked to increased risk of CSF diversion, limited data exist on the predictive value of postoperative fetal imaging.

**Study Design**: This was a nested case‐control study in a cohort of patients who underwent fetal surgery (open or fetoscopic) for ONTD at a single center between 2020 and 2024. Cases were those requiring CSF diversion within the first year of life; controls did not require diversion. Pre‐ and postoperative prenatal ultrasound images were reviewed, and growth trajectories of relevant biometric markers were analyzed. Logistic regression and receiver operating characteristic (ROC) curves were used to evaluate predictors of postnatal CSF diversion.

**Results**: Thirty patients met inclusion criteria; 27% had fetoscopic repair, and 30% required CSF diversion. Pre‐ and intraoperative factors, including lesion type, preoperative LV width, gestational age at repair, and surgical approach, did not differ between groups. Preoperative LV growth rates were similar between groups, but postoperative LV growth rates were significantly higher among patients ultimately requiring diversion (0.58 vs. 0.16 mm/week, *p* < 0.01). While resolution of the “banana sign” was more common in those avoiding diversion, cerebellar growth rates were similar between groups. Postoperative LV growth rate predicted need for CSF diversion with an area under the ROC curve of 0.88 (95% CI 0.74–1.00); the optimal cutoff for prediction of CSF diversion was 0.32 mm/week which had a sensitivity of 100% and specificity of 75%. Each 1 mm/week increase in postoperative LV growth was associated with a 23‐fold increase in odds of CSF diversion (OR 22.9, 95% CI 1.46–359.3).

**Conclusion**: Postoperative LV growth rate after fetal ONTD repair is a strong predictor of CSF diversion. Serial fetal imaging to assess LV trajectory may improve prenatal counseling and guide postnatal neurosurgical surveillance.

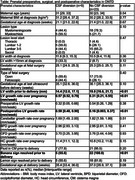

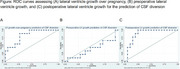

**2:45 PM  –  3:00 PM**

## 36 | **Two Year Outcomes in Premature Infants Randomized to Umbilical Cord Milking Versus Delayed Cord Clamping**

Anup Katheria^1^; Jeff M. Szychowski^2^; Nicole Wilson^3^; Kathy Arnell^4^; Yvonne Vaucher^4^

On behalf of the Premod2 Study Group

^1^Sharp Mary Birch Hospital for Women & Newborns, Sharp Mary Birch Hospital for Women & Newborns, CA; ^2^Department of Biostatistics, University of Alabama at Birmingham, Birmingham, AL; ^3^Department of Biostatistics, University of Alabama at Birmingham, Burmingham, AL; ^4^Sharp Mary Birch Hospital for Women & Newborns, San Diego, CA

**Objective**: The PREMOD2 trial showed umbilical cord milking (UCM) vs delayed cord clamping (DCC) increases severe intraventricular hemorrhage in extremely preterm infants (23–27 weeks) but not in very preterm infants (28–32 weeks). We compare post‐discharge neurodevelopmental (ND) outcomes at 2 years corrected age (CA) in children from PREMOD2.

**Study Design**: ND outcomes of preterm infants born 23 0/7–32 6/7 weeks were assessed with Bayley Scales of Infant Development 3^rd^ (BSID‐3) or 4^th^ (BSID‐4) ed. Assessors were blinded to treatment allocation. Motor and neurosensory impairments were ascertained by in‐person exams, parent phone interviews or chart abstraction. BSID scores were compared between study groups and strata and evaluated at a 2‐point non‐inferiority margin. Mild, moderate and severe ND impairment (NDI) were classified using standard definitions of cognitive impairment, abnormal gross motor classification score, vision or hearing impairment.

**Results**: Of 1201 infants enrolled in the original study there were 40 deaths (20 per group). Of the remaining infants, 996 (86%) had some information on ND outcomes at 22‐26 months CA. Demographics in those assessed by in‐person BSID‐3 or 4 (UCM, *n* = 436 and DCC, *n* = 438) groups were similar. Children in both groups had similar cognitive and language scores, though differences exceeded the non‐inferiority margin (Table 1). UCM motor scores were marginally lower. There were no differences in hearing loss (1.7% vs 2.1%, *p* = 0.66), visual impairment (4.7% vs 3.5%, *p* = 0.38), cerebral palsy (4.3% vs 3.7%, *p* = 0.64), mild NDI (12.2% vs 12.6%, *p* = 0.86), or moderate to severe NDI (8.3% vs 8.2%, *p* = 0.98) in UCM vs DCC, respectively. Rates of normal BSID scores by gestational age strata are in Table 2.

**Conclusion**: In PREMOD2, rates of NDI assessed by BSID at 2 years CA did not differ substantially between UCM and DCC groups. Combined with previously reported similar short‐term outcomes in infants 28–32 weeks, these data suggest UCM in this group is a feasible, no‐cost intervention without longer‐term neurodevelopmental risks of cord milking.